# Printing Insecurity? The Security Implications of 3D-Printing of Weapons

**DOI:** 10.1007/s11948-014-9617-x

**Published:** 2014-12-18

**Authors:** Gerald Walther

**Affiliations:** Faculty of Life Sciences, Institute for Science Ethics and Innovation, University of Manchester, Stopford Building, Oxford Road, Manchester, M13 9PT UK

**Keywords:** Security policy, 3D-printing, Arms control

## Abstract

In 2013, the first gun printed out of plastic by a 3D-printer was successfully fired in the US. This event caused a major media hype about the dangers of being able to print a gun. Law enforcement agencies worldwide were concerned about this development and the potentially huge security implications of these functional plastic guns. As a result, politicians called for a ban of these weapons and a control of 3D-printing technology. This paper reviews the security implications of 3D-printing technology and 3D guns. It argues that current arms control and transfer policies are adequate to cover 3D-printed guns as well. However, while this analysis may hold up currently, progress in printing technology needs to be monitored to deal with future dangers pre-emptively.

## Introduction

Matthew Meselson, head of the Harvard Sussex Program on Chemical and Biological Warfare Armament and Arms Limitation, once stated that “every major technology—metallurgy, explosives, internal combustion, aviation, electronics, nuclear energy—has been intensively exploited not only for peaceful purposes but also for hostile ones” (Meselson 2000). On May 3rd, 2013, another novel technology has joined this club—3D printing. On this day, Cody Wilson, founder of Defense Distributed, shot the first 3D-printed plastic gun, called the ‘Liberator’. In the wake of this demonstration that it is possible to print a working plastic gun, the media has intensively covered this topic in the form of interviews with Cody Wilson and various law enforcement agencies all over the world as well as reports on the weapon’s functionality and its dangers. It is the purpose of this article to critically assess the reports that have come in on the dangers that are posed by these weapons to human and international security, as well as their implications for international disarmament treaties such as the arms trade treaty (ATT).

## Some Facts About the ‘Liberator’

The ‘Liberator’ is a one bullet handgun. At first glance, there is nothing remarkable about the ‘Liberator’. It looks slightly odd compared to a regular handgun with its stubby barrel. When disassembled, it consists of 16 parts, 15 of which can be printed by a 3D printer. The only non-plastic part thus far is a common hardware store nail, which is used as the firing pin. The blueprints necessary to print out the gun had been made available online for free by Defense Distributed on the 3rd of May, 2013. On the 8th of May, 2013, The US Department of States, Bureau of Political-Military Affairs, Office of Defense Trade Control Compliance, sent a letter to Wilson asking him to take down a variety of data files because it had to be established if they violated the Export Control Act (Greenberg 2013a). The files for the ‘Liberator’ were among these data files. Until the removal of the files, they had been downloaded about 100,000 times in these first 2 days (Greenberg 2013b). Files were also stored on Mega.com, a file sharing network run by Kim Dotcom in New Zealand. Kim Dotcom removed the files as well because he considered them to be a ‘threat to the security of the community.’ (*Ibid*.) Of course, even now it is still possible to find the blueprints at various other file sharing websites, e.g. the Pirate Bay, so it is practically impossible to remove them from internet circulation. According to Haroon Khalid, a developer working with Defense Distributed, the ‘Liberator’ has been predominantly downloaded in the US, Spain, Brazil, Germany, and the UK (*Ibid.*).

But does the gun actually work? The release of the blueprints was accompanied by a YouTube video of Cody Wilson shooting the gun for the first time. The gun has also been tested by several law enforcement agencies all over the world (Kantchev 2013a). Experts at the Austrian Interior Ministry concluded after their tests that the gun is indeed a deadly weapon (*Ibid.*). However, they also noted that they had to change the barrel after each shot was fired (*Ibid.*). In the US, the Bureau of Alcohol, Tobacco, Firearms and Explosives (ATF) printed several versions of the gun (Reilly 2013). The performance of the gun depended heavily on the plastic used in the creation. The use of plastic from the company Visijet resulted in the explosion of the gun during firing while using acrylonitrile butadiene styrene plastic the gun performed like a regular gun, albeit being less powerful but still enough to penetrate several inches of soft flesh as well as the human skull (*Ibid*.). Police have thus argued that the gun might be used by assassins who only need to fire one round. The reason behind this logic is that the gun is very difficult to detect as it only requires a small iron pin as the firing mechanism and therefore would not be easily spotted by metal detectors. A reporter of the UK Mail on Sunday took the gun on board of the London–Paris train and assembled it in the toilet (Murphy and Myers 2013). However, he neither took the metal firing pin nor any bullets onto the train due to possible legal ramifications. The sensationalism behind the story is thus quite exacerbated as any real assassin or terrorist would have to smuggle these two additional items on board as well. While the small metal firing pin may be easily concealed or maybe in the future could be made from plastic as well, smuggling bullets through metal detectors is a more difficult challenge. Attempts at making printed bullet have taken place but these perform very poorly and only work as shotgun slugs (Kleinman 2013). In addition, even these slugs needed additional weight in the form of a lead ball (*Ibid.*).

Since the introduction of the Liberator, the 3D gun printing community has produced other weapons as well. On 23 September 2013, a user posted the files for the printing of a .22 LR semi-automatic gun on FOSSCAD, a data sharing website for 3D-printing designs (Tuccille 2013) (the original file on FOSSCAD has subsequently been removed). While the gun requires a firing control group, a firing pin, and buffer spring from an AR-15 and a metal insert for case extraction, all other parts are made from plastic. The user, Proteus, also argued that the firing control group could also be made out of plastic if the gun is made as a fully automatic one. To date, no videos of an attempt to shoot the gun are available online.

On 6 November 2013, US Company Solid Concepts released a video that showed the first 3D metal-printed gun—a M1911 (Solid Concepts 2013). The Company claims that the gun successfully fired more than 600 rounds without any malfunctions. At the presentation of the printed gun, there was hardly any concern as metal printers are extremely expensive—most of them were and still are in use for industrial rather than home purposes and thus cost hundreds of thousands of dollars. However, on 26 November 2013 the Michigan Technological University newsletter published an story that described how a university group led by Joshua Pearce built a 3D metal printer using components and materials that cost less than 1,500 US$ (Goodrich 2013). The accompanying article published in IEEE Access describes how to assemble the printer (Anzalone et al. 2013). While the printer may not necessarily produce the same results in terms of quality as industrial metal printers, it may not be farfetched to argue that they are going to increase in quality in the near future.

The previous examples highlight that the ability to produce a gun or weapon using 3D printing technology is assisted by three developments: first, individuals interested in creating guns are developing better models for guns and weapons. In addition, they may develop very novel and unique types of weapons that look and work very different from today’s weapons. Second, advances in chemistry will lead to stronger plastics, e.g. which type of plastic the ATF used in printing the Liberator was very important, and it may be possible in the future to print both the firing pin and bullets from plastic, even though the latter might be vastly more difficult. And third, advances in 3D printers themselves could have substantial impacts in determining the types of weapons and their quality that can be made by individual users at home.

## National and International Regulations

Given the novelty of 3D printed weapons, it is hardly surprising that the current academic literature on the topic is limited. Yet, a very detailed analysis of the implications of 3D printed weapons for the US legal systems, specifically with reference to the Second Amendment, had been given by Jensen-Haxel (2012). While the publication and discussion in 2012 does not include the latest development, specifically the introduction of the Liberator, it nevertheless anticipated the events to come and analysed them accordingly. What Jensen-Haxel did not take into consideration in his article were current US arms trade regulations as well as potential security questions these weapons could pose. Jensen-Haxel concluded in his article that current efforts to curtail the supply side of weapons will be undermined if consumers can simply print out whatever components they need via a 3D printer. Currently, it is only the frame that is actually regulated by law once a weapon is disassembled (The frame, or receiver, is the part of a gun that holds all pieces together and generally contains the operational mechanism. It also tends to hold both the trigger and the magazine). Yet, before the publication of the Liberator, Defense Distributed had already published blueprints of how to make an AR-15 semi-automatic lower receiver, which is part of the frame, out of plastic (the frame of an AR-15 consists of an upper and lower receiver—under US law, the lower receiver, which includes the serial number, is the legally controlled part). Their video of a semi-automatic rifle with the plastic frame is available on the Internet and Defense Distributed claims that the frame works without any problems (Farivar 2013). Jensen-Haxel argues that any attempts at regulation of additive manufacturing would hamper economic and technological progress and would thus not be in the interest of the US. In his view, given the potential future lack of control on the supply side, the US government should rather place ‘new emphasis… on improving impoverished communities and re-examining our [US] drug policies in an effort to abrogate black markets’ (Jensen-Haxel 2012).

While the right to manufacture arms, within limits, e.g. no automatic weapons, in your own home may be protected by the Second Amendment within the US, the case is more difficult once it reaches the international stage. Defense Distributed were asked to take down the blueprints of the Liberator and other items they had designed because they did not have an arms export license and not because they were not allowed to produce a weapon for their own private use. Of course, this right to produce a firearm for personal use may just be unique to the US. So what is the situation in the other top five countries, i.e. Spain, Brazil, Germany and the UK, in which the Liberator file was downloaded most often? In Spain, ‘the Constitution has reserved to the State the exclusive competence on issues related to the production, trade, possession, and use of firearms and explosives’ (Library of Congress 2014a). In Brazil, ‘the production of armaments (*material bélico*) and the arms trade are regulated by the federal government. The Penal Code criminalizes conduct involving, inter alia, the handling of materials for the production of arms devices’ (Library of Congress 2014b). In Germany, production of arms is regulated by law and one needs a *Waffenherstellungserlaubnis* (‘permit for the production of arms’) to do so (Bundesministerium der Justiz und für Verbraucherschutz 2014). In the UK, the production of firearms without a license is prohibited by the 1968 Firearms Act. But even though 3D guns are therefore already covered, the UK government ‘updated its rules to prohibit the manufacture, sale, purchase and possession of them’ (Berry 2013). In terms of the dissemination of blueprints that enable the printing of a 3D gun like the Liberator, Spain, Germany and the UK have national laws that comply with the 2008 EU Common Position on arms exports. The Common Position applies to all items on the EU Common Military List. With regard to the publication of blueprints for the Liberator or similar weapons, the Military List under ML 22 b. 2. includes: ‘“Technology” “required” for the “development” and “production” of small arms even if used to produce reproductions of antique small arms’ (EU Council 2012). Under the Common Position, anyone disseminating blueprints for a 3D gun would therefore have to apply for an export license permit. As there is no definite end-user when disseminating blueprints over the internet, it is highly unlikely that a license would be granted. But of course, it is precisely this sort regulation that Defense Distributed founder and self-proclaimed crypto-anarchist Cody Wilson wanted to get around by publishing the blueprints in the first place (Cadwalladr 2014).

Besides national and EU law, there are also international agreements such as the ATT that have an impact on the distribution of printed weapons and their blueprints. The ATT was adopted on 2 April 2013 and will enter force on 24 December 2014. Thus far it has been signed by 122 States Parties and ratified by a further 54, which leaves 17 countries outside of the treaty. In terms of its scope, the ATT covers small arms and light weapons as well as arms components and thus applies to any printed gun like the Liberators as well as to the printed AR-15 control group. However, it does not cover any technology transfer and therefore does not apply to blueprints for 3D weapons. As with other UN arms control conventions, the ATT does not constitute a legal text, ratifying the convention requires the adoption of relevant national legislation.

Besides the ATT, the ‘Wassenaar arrangement on Export Controls for Conventional Arms and Dual-Use Goods and Technologies’ also covers printed weapons. The Wassenaar arrangement was developed to increase regional and international stability and security. It is composed of 41, mostly industrialized, countries. It works similar to the ATT in that it requires participating countries to adopt national legislation and develop effective export controls. In addition to covering the export of physical components, it also includes technology transfer and thus applies to blueprints of 3D weapons.

In conclusion, national laws that deal with the production of weapons directly apply to the production of printed weapons as well. Of course, it may be more complicated from a practical point of view to control the printing of a gun compared to manufacturing a metal gun. Similarly, with regard to the transfer of weapons, both national law as well as international agreements that regulate the transfer of arms also apply to 3D printed ones as well as their blueprints. While the actual control of transfer of 3D arms and component does not provide any novel problem to national agencies, the regulation of blueprints appears nearly impossible. The question is thus if and to what degree the availability of blueprints and the associated potential for an increase in gun ownership will present a challenge to national and human security.

## National and Human Security Implications of 3D Weapons

### Crime and Gun Ownership

As described above, law enforcement agencies were highly interested in 3D guns as they perceive them to be a threat to security in their respective countries. The fear comprises two elements: first, 3D guns make it easier for everyone to acquire a gun and thus increase the danger to police officers as well as civilians; and second, 3D guns are difficult to detect so they might be ideal for assassination of terrorist attacks. With regard to the first assumption, the rationale is that a larger number of weapons will result in an increase in gun-related crimes and deaths. While this hypothesis is a hotly contested topic, statistical comparison seem to support that a higher availability of guns puts men and women at a higher risk of homicide, particularly firearm-related homicides (Harvard Injury Control Research Centre 2014; Hepburn and Hemenway 2004). This argument runs counter to the claim by Cody Wilson that more guns will make people safer as guns level the playing field and allow citizens to better protect themselves. Further information on the costs and benefits of gun ownership can be taken from the Small Arms Survey (SAS), which is an independent research project located at the Graduate Institute of International and Development Studies in Geneva, Switzerland and provides ‘an annual review of global arms issues such as production, stockpiles, brokering, legal and illicit arms transfers, the effect of small arms, and national, bilateral, and multilateral measures to deal with the problems associated with small arms’ (Small Arms Survey 2014). It produces reports on stockpiles of small arms, civilian and state possession, as well as armed violence. In its 2013 report the SAS points out the danger, specifically to women, of private gun ownership. Women are specifically endangered from intimate partner violence (IPV), e.g. murder-suicide by their partner. Specifically in countries with low homicide rates women are primarily in danger from being harmed by IPV. In a survey of 16 European countries, the SAS found that 43 % of femicides are committed by spouses or ex-spouses. The SAS also found that in countries with high prevalence of guns in households, the risk of murder-suicide with firearms is increased. The SAS concludes that the risk of keeping a gun at home outweighs any benefits (Small Arms Survey 2013; chapter 2). An increase in gun ownership due to 3D printing may thus be particularly harmful to women.

On the other side, acquisition of 3D printed guns may not necessarily imply that individual gun availability increases. In its survey of civilian gun ownership, the SAS reported in 2007 that there are about 650 million guns in civilian possession (without the US the number drops to 380 million) (Small Arms Survey 2007). While guns tend to be more abundant in the aftermath of a violent civil war, the SAS found that there is a positive relationship between wealth and gun possession. Countries with higher income tend to have more guns. Given that it is yet very expensive to obtain a 3D printer, guns will most likely be printed in rich countries, which already have more guns available than poorer countries. It is yet too early to give an account of where 3D printed guns will appear but one hypothesis is that 3D guns will be printed by those that already have access to ‘normal’ guns and just want to have one for its novelty rather than for its practical use. This novelty aspect may have a worrying side-effect in attracting the attention of adolescents that want to print one for the sake of it being ‘new’ and ‘cool’. This may lead to an increase in gun accidents if they are careless—specifically given that plastic guns are less reliable and thus more dangerous than their metal counterparts. As the US ATF found out, some printed guns may explode upon firing. It is yet too early to test this hypothesis. However, going back to the issue if printed guns pose a new challenge to combating gun-crime, if one is intent on using a gun for crime, given that only 79 million civilian guns are actually registered, which is just 9 % of the total suspected guns, it may actually be easier to gain access to an unregistered metal gun, which is for now also more efficient than a ‘Liberator’.

### Law Enforcement Concerns

While 3D guns may thus only be regarded as a curiosity item for a gun collector, there are some elements to 3D guns that do pose problems to law enforcement. While unlicensed guns are in circulation, one needs to get into contact with a third party to acquire one. In contrast, printing a 3D gun can be done in complete secrecy. And the gun can also be easily destroyed by melting the plastic again, which would leave no trace of its existence. So while there is a possibility for the police to trace weapons and based on bullet identification even match a gun to a certain bullet and thus a crime scene, this option is unavailable for 3D gun crimes. Of course, given the rather unique shape of the Liberator and its components, it may be possible for law enforcement to conclude that the gun used was indeed a plastic gun. However, with the ease of destruction of a plastic gun, police can only search for 3D printers. However, if the culprit also deleted any cache and buffer files on the printer and the computer, and erased (and/or hid using tools like ‘The Onion Router’) his internet activities when downloading the files, it would be impossible link a suspect to a crime by way of gun use and ownership.

In addition to the problem of tracking the gun, a 3D printed plastic gun presents problems primarily because it is made out of plastic. Yet, as argued above it is still impossible to print any actual bullets out of plastic. In addition, the pin still needs to be made out of metal as well. While it is therefore possible to smuggle it onto a train like the Mail on Sunday reporter managed to do, or even onto an airplane, the gun is useless without metal parts, which can be spotted with standard metal scanner that are already in place in sensitive locations such as airports. It may also be questioned how effective a gun like the Liberator would even be in the case of for example taking over an airplane. The gun can only fire one bullet and then most likely the barrel has to be replaced, as the Austrian police forces discovered (Kantchev 2013b). By comparison, a knife might be more dangerous as it can be used several times. For example, if a terrorist was forced to shoot the gun in an airplane hijack, his leverage over the other passengers would be completely gone.

For a metal 3D printed gun, the only difference to a normal gun is that the owner can produce it on his/her own instead of having to purchase it from someone. All other aspects, e.g. bullet identification and bypassing security controls, are exactly the same as for ‘normal’ guns. However, in terms of development, it is possible that civilian arms using metal 3D printing will in the future match those of military forces in terms of power. Currently, full-automatic assault rifles and similar weapons are only available to the military in most countries, except if they have recently suffered from a civil war and demilitarization has not taken place or been successful. 3D metal printing might shift this imbalance.

### National Security: Large Scale Weapon Production

Beyond individual interest in the acquisition of printed guns—whether plastic or metal—there is reason for concern that the technology will undermine any attempts to regulate and control the illicit arms trade, specifically with regard to small arms. While the Arms Trade Treaty covers a variety of weapons, it was hoped that it would specifically reduce the illegal trade in small arms and light weapons, as it is those are the ‘real weapons of mass destruction’ because they cause 1,300 deaths every day (Small Arms Survey 2001, p. 1). The impact of small arms violence does not only include the deaths themselves but it adversely affects the economic situation of those affected by small arms violence. Non-fatal injuries put a heavy economic burden on individuals and their family due to poor healthcare as well as indirect costs because of eventual disabilities and therefore lost productivity. In addition, the threat to health and safety decrease the overall standard of living as well as mental health. On a state level, high levels of violence require higher spending on police and military, thus reducing the ability to provide other forms of services, such as for example healthcare or creating incentives to increase investment. But even if 3D printing could theoretically undermine treaties such as the ATT, will this actually have an impact on security? As the SAS has pointed out, there are currently about 380 million small arms in circulation worldwide (excluding the US). Even with the introduction of regulations on arms trade, these weapons are still out there and it is unlikely that any trade regulation will prevent their circulation. Fragile states, e.g. those that have recently come out of a civil war or where the government does not have control over its entire territory, hardly have the capacity to disarm or control the weapon transfer from insurgent groups or large scale criminal organizations. 3D printing may therefore not change the ability to acquire weapons. While it may be argued that it is easier because these actors could simply acquire weapons components excluding the frame (or more specifically the lower receiver, which is the only component that is actually regulated and registered domestically in the US for example) and then print out the remaining parts, thus not violating arms export controls, arms components, not just the frame, are included in the ATT. However, while 3D printing of guns may not significantly increase the challenge to reduce the number of unlicensed weapons, e.g. there are around 100 million AK-47 in circulation worldwide, it could lead to the development of novel weapons. For example, 3D plastic printers could be used to print landmines or IEDs, which might be harder to detect using metal detectors. Metal 3D printers may also help armed groups to acquire better weapons and equipment and to repair existing ones, even though at the moment they are still too expensive and impractical.

## Conclusion

The purpose of this article was to give an overview of security implication of current 3D printing capabilities. While it has been argued that higher availability of guns leads to a higher risk of homicide, it does not necessarily follow that 3D printed guns will increase the availability of guns. They may just be seen as ‘novel’ items by weapons collectors and enthusiasts. The inability to print bullets as well as the limited effectiveness of the Liberator, i.e. a one shot device that is rather weak and inaccurate, also reduces the risk that it could be used for terrorist and criminal purposes. Normal weapons are already available in abundance worldwide, particularly in countries with higher wealth as well as in post-conflict countries, which reduces the incentive to acquire a gun like the Liberator. In terms of metal 3D printing the technology is still very expensive and thus not accessible to the average person, and nearly impossible to acquire for an armed group in a third world country. However, given the general decline in costs of technology over time, it is necessary to think about the implication of 3D printing now before the technology becomes widely available. But is it possible to regulate 3D printing? One suggestion has been to change the internal coding of the 3D printer to make it unable to print out a gun. This idea seems hardly feasible though. How can the printing software understand what a gun is? Also, if I want to print out a plastic toy gun for my child to use for Halloween, why am I not allowed to do so? Furthermore, printers would have to be continuously updated to know what new design they are not allowed to print. This seems hardly feasible. But even if this idea were to work, what would stop someone from hacking the software? The computer gaming and the music industry, despite their best efforts, have never managed to develop a working security measure to prevent illegal copies of their software or music. If any modification of the printers is therefore unfeasible, this leaves the production components, i.e. controlling the plastics. However, it will certainly harm the market if users have to apply to an agency and give detailed information on how they want to use a specific plastic that they want to acquire. Another option is to control and restrict the blueprints to at least make it more difficult for users to print the weapon. However, as the Liberator example has shown, once a file is out for only 3 days and probably less, it is available online for ever. A final option is self-regulation. The 3D printing community may want to exercise self-restraint and not publish blueprints of weapons or components. In line with regulation of blueprints, this does not prevent a determined user from designing a gun using a CAD program in their own, but at least it would not be as easy as simply downloading a file and then printing it out. However, people like Cody Wilson actually want weapons to be available everywhere for everyone, without any consideration for the potential harm this unregulated distribution can have. But in the absence of regulatory alternatives, it may be helpful to engage the 3D printing community in a rational dialogue about the potential implications of their research. Possibly they could adopt a code of conduct. This approach is not unprecedented. For example, the FBI and the Do-It-Yourself (DIY) Biology community have worked together to raise awareness among the DIY community of the potential security concerns of DIY Biology activities (Lempinen 2011; You 2010). A similar collaboration might be useful to mitigate security concerns of 3D printing as well. In addition, one specific aspect of plastic guns should be carefully monitored: the development of plastics that could actually function as bullets. This development would require a re-analysis of the security implications of 3D printing. In order to deal with the ensuing novel risks, it would be advisable to now engage in a continuing dialogue with the chemical industry to raise awareness of the danger that plastic bullets could create. In this way, a solution or approach could be formulated before the genie is out of the bottle—unlike the case of the ‘Liberator’.

In general, at the moment 3D printing does not create any novel security concerns. Weapons, including a huge portion of unlicensed ones, are quite abundant worldwide and pose a more problematic security challenge than any printed gun out of plastic. Current national and international legislation are also not circumvented by 3D printing and there is no need to develop new international treaties just for printed guns.
